# Divergent Pathways in COS-7 Cells Mediate Defective Internalization and Intracellular Routing of Truncated G-CSFR Forms in SCN/AML

**DOI:** 10.1371/journal.pone.0002452

**Published:** 2008-06-18

**Authors:** Melissa G. Hunter, Morgan McLemore, Daniel C. Link, Megan Loveland, Alexander Copelan, Belinda R. Avalos

**Affiliations:** 1 Pulmonary, Allergy, Critical Care and Sleep Medicine, The Ohio State University, Columbus, Ohio, United States of America; 2 Davis Heart and Lung Research Institute, The Ohio State University, Columbus, Ohio, United States of America; 3 Winship Cancer Institute, Emory University School of Medicine, Atlanta, Georgia, United States of America; 4 Division of Bone Marrow Transplantation and Stem Cell Biology, Washington University, St. Louis, Missouri, United States of America; 5 Division of Hematology/Oncology, The Ohio State University, Columbus, Ohio, United States of America; 6 Johns Hopkins University, Baltimore, Maryland, United States of America; Ordway Research Institute, United States of America

## Abstract

**Background:**

Expression of truncated G-CSFR forms in patients with SCN/AML induces hyperproliferation and prolonged cell survival. Previously, we showed that ligand internalization is delayed and degradation of truncated G-CSFR forms is defective in patients with SCN/AML.

**Methodology/Principal Findings:**

In this study, we investigated the potential roles of dileucine and tyrosine-based motifs within the cytoplasmic domain of the G-CSFR in modulating ligand/receptor internalization. Using standard binding assays with radiolabeled ligand and COS-7 cells, substitutions in the dileucine motif or deletion of tyrosine residues in the G-CSFR did not alter internalization. Attachment of the transferrin receptor YTRF internalization motif to a truncated G-CSFR form from a patient with SCN/AML corrected defective internalization, but not receptor degradation suggesting that receptor internalization and degradation occur independently via distinct domains and/or processes.

**Conclusions:**

Our data suggest that distinct domains within the G-CSFR mediate separate processes for receptor internalization and degradation. Our findings using standard binding assays differ from recently published data utilizing flow cytometry.

## Introduction

Granulocyte colony-stimulating factor (G-CSF) critically regulates neutrophil numbers by binding to the G-CSF receptor (G-CSFR) to generate signals that maintain a homeostatic balance between myeloid cell survival, proliferation, and differentiation [1]–[4]. Despite continued progress in our understanding of the signaling pathways that are activated by the G-CSFR to transduce mitogenic signals, little is currently known about the mechanisms by which the G-CSFR downregulates proliferative signaling.

For those growth factor receptors that have been studied, ligand-induced receptor internalization has been shown to critically control cellular responsiveness [5]–[9]. Following ligand binding, most receptors are recruited to clathrin-coated pits where they are endocytosed then internalized and either recycled back to the cell surface or degraded within intracellular compartments. Sorting signals within the cytosolic tails of the receptors have been identified that direct trafficking of the receptors [10]. Tyrosine-containing and dileucine-containing regions are two characteristic motifs responsible for endosomal-lysosomal targeting of receptor proteins. Receptor internalization and degradation serve to attenuate receptor signaling by rapidly down-modulating or decreasing the number of receptors on the cell surface to protect cells from over-stimulation. Receptor trafficking also controls the specific signaling pathways triggered by activated receptors and the intensity of signaling [9], [11], [12].

Abnormalities in both receptor oligomerization and trafficking have been shown to alter normal receptor function and ligand sensitivity and to contribute to human diseases [13]–[16].Our laboratory previously reported that ligand internalization and receptor degradation are impaired leading to sustained cellular activation and enhanced cell survival and proliferation in patients with severe congenital neutropenia (SCN) transforming to (AML) in which a truncated G-CSFR is expressed along with the wild type form [17]. Our data were subsequently confirmed by others in both *in vitro* and *in vivo* studies [18], [19].

The precise defects responsible for abnormal degradation and modulation of surface expression of the G-CSFR in SCN/AML remain unknown. In this study, we have examined the roles of known internalization motifs within the cytoplasmic domain of the G-CSFR to determine whether they modulate G-CSFR internalization and degradation. Our data suggest that the G-CSFR utilizes distinct and separate processes for receptor internalization and degradation as a mechanism for regulating receptor surface expression and recruitment of specific signaling pathways.

## Methods

### Reagents

Cell culture reagents, restriction enzymes, and oligonucleotides were purchased from Invitrogen Inc. (Carlsbad, CA). G-CSF was a generous gift from Amgen Inc (Thousand Oaks, CA). [I^125^] G-CSF (>800ci/mmol) and Pro-mix [^35^S] *in vivo* cell labeling mix (>1000ci/mmol) were obtained from Amersham (Arlington Heights, IL). All other reagents were purchased from Sigma Chemical Co (St. Louis, MO) unless otherwise indicated.

### DNA constructs

The construction of pCDNA3.1-WT, the wild-type human Class I G-CSFR cDNA (generously provided by Dr. A. Larsen, Seattle, WA) and pCDNA3.1-Δ716 have been previously reported [17]. Tyrosine to phenylalanine (Y→F) mutations at amino acid positions 729 and 764 of the WT G-CSFR were introduced by site directed mutagenesis and the corresponding G-CSFR forms were designated Y729F and Y764F, respectively. To generate Y729F and Y764F, an A to T point mutation at nt. 2465 or 2525 was introduced, respectively, in the WT-G-CSFR cDNA by overlap extension PCR [17]. The oligonucleotides used to generate these mutants were: forward primer Y29F F1 (5′-GGCACCCAGCCTGCCCAAAAAGTACTTGATC-3′), forward primer Y764F F2 (5′-GGCCAGGGCACTTTCTCCGCTGTGA-CTCCACTC-3′), reverse primer Y729F R1 (5′-GCCCAGCAGCGATCAAGTACTTTTTGGGCAGCT -3′), and reverse primer Y764F R2 (5′-GGAACCAGAAATTCTCAAAGCTTTTGGGG CTGG-3′); the underlined nucleotides indicate the positions of the point mutations. To generate Y744F, primer F3 containing a 5′ restriction site for *Bam* HI and corresponding to nts. 2252-2268 of the WT-G-CSFR cDNA was used in conjunction with the Y729F R1, to amplify WT-G-CSFR cDNA. In a separate PCR reaction, WT-G-CSFR cDNA was amplified with Y729F F1 primer and primer R3 that was designed to contain a *Xho* I restriction site and corresponded to nts. 2581-2596 of the WT-G-CSFR cDNA. The products from the two initial PCR reactions were combined and amplified by PCR using the external primers F3 and R3. The PCR product was digested with *Xho* I and *Bam* HI and then subcloned into the *Xho* I and *Bam* HI sites of pBluescript SK^+^. An internal 280bp fragment in the cloned product was excised from pBluescript SK^+^ by *Cfr*10I and *BstE* II digestion, gel purified, and cloned into WT cDNA replacing WT sequences. This plasmid was designated pCDNA3.1-Y729F.

To generate the Y764F mutant, primer pairs Y764F F2/R3 and F3/Y764F R2 were used in initial PCR reactions and the resulting PCR products further amplified with oligonucleotides F3 and R3. The same cloning strategy was used to generate pCDNA3.1-Y764F.

Point mutations at amino acid residues 753, 754, or both 753 and 754 of the WT G-CSFR were generated using the Transformer Site-Directed Mutagenesis kit (BD Clontech, Palo Alto, CA) and designated L753A, L754A, and L753/754A G-CSFR. Briefly, ssDNA was generated from plasmid DNA containing the WT G-CSFR cDNA by incubation at 100°C for 5 min. Mutagenic oligonucleotides specific for the G-CSFR, and vector mutagenic oligonucleotide (5′-PO_4_-CTCTGGGGATCGATATGACCGACC-3′) which changes a *Sfu*I restriction site to *Cla*I restriction site (underlined) were annealed to the ssDNA template then incubated with reaction buffer containing T4 DNA polymerase and T4 DNA ligase for 90 min at 37°C. The following 5′-phosphorylated oligonucleotides were used to generate L753A (5′-TGACTCCACTCAGCCGGCTTTGGCGGGCCTCAC -3′), L754A (5′-TGTGACTCCACTCAGCCTCTAGCCGCGGGCCTC-3′), and L753/754A (5′-TGTGACTCCACTCAGCCTGCCGCGGCGGGCCTCAC-3′). In addition to the Leu to Ala substitutions (underlined), silent mutations were made to generate a *Nae*I restriction site in L753A and a *Ksp*I restriction site in both the L754A and L753/754A cDNA. The DNA was digested with *Sfu*I and transformed into BMH 71-18 *mut*S competent cells, which is a repair defective strain of E. *coli* which preferentially selects mutated DNA for replication. The DNA was isolated and restriction digested with *Sfu*I and transformed into DH5α E. *coli* and selected for ampicillin resistance. The introduced mutations were confirmed by restriction endonuclease digestion and sequencing.

The QuikChange Site-Directed Mutagenesis kit (Stratagene, La Jolla, CA) was used to construct the Δ716-YTRF G-CSFR form in which the YTRF internalization sequence from the transferrin receptor was inserted 5′ to the stop codon in Δ716 cDNA. Briefly, cDNA corresponding to Δ716 G-CSFR form subcloned into pBluescript SK+ was denatured and incubated with mutagenic sense (5′-GCAGTTTCCACCTATACCCGGTTCTAGCC-CCAATCCC-3′) and antisense (5′-GGGATTGGGGCTAGAACCGGGTATAGGTGGA-AACTGC-3′) oligonucleotides containing the sequence encoding for amino acid residues YTRF (underlined) and *Pfu* Turbo DNA polymerase. Nonmutated parental DNA which is methylated was digested using methylation sensitive restriction enzyme, *Dpn*I. Supercompetent cells were transformed with the mutated nicked dsDNA for repair and amplification. Introduction of the YTRF sequence introduced an additional *Nci*I restriction site allowing confirmation of the addition of YTRF sequence by restriction digestion. cDNA was subcloned into pCDNA3.1 and sequenced to confirmed the addition of YTRF to the Δ716 G-CSFR.

### Transfections and cell culture

COS-7 cells were grown in DMEM supplemented with 10% FBS and were transiently transfected by calcium phosphate precipitation with plasmid DNA, as previously described [17]. An equivalent amount of plasmid DNA was used for single transfections in COS-7 cells.

### Internalization studies

Ligand internalization was performed as previously reported [17]. Briefly, cells were incubated with 500pM [I^125^] G-CSF for 2 h at 4°C. Internalization of radiolabeled ligand was examined by temperature shifting to 37°C for varying times from 30 to 120 mins. Following incubation at 37°C, the cells were incubated for an additional 2 h at 4°C. To remove unbound ligand, cells were washed in cold PBS containing 1mM MgCl_2_, 0.1mM CaCl_2_, and 0.2% BSA. Surface-bound [I^125^] G-CSF was removed by incubation with 0.5M NaCl (pH1.0) for 3 mins followed by washing in cold PBS solution. The acid strip solution and wash were both collected to determine bound ligand. Internalized ligand was quantified by lysis of the cells with 1M NaOH for 1min. The data were expressed as percent internalization over time [20]. Analysis of specific binding was also performed in the presence of excess cold ligand as previously described [17], [21], [22].

### Receptor degradation

Transfected COS-7 cells grown to confluency in T75 flasks were incubated with short-term labeling media (RPMI 1640 media containing 10% dialyzed FBS without methionine and cysteine) for 15 mins at 37°C. Cells were metabolically labeled with [^35^S] Cysteine/Methionine Pro-mix at 0.15mCi/ml in short-term labeling media for 1 h at 37°C as previously described [10], [20], [23]. The cells were washed with incubation media (RPMI 1640 containing 10% FBS, 1nM G-CSF, and unlabeled methionine and cysteine) and incubated for varying times, then washed once in PBS, scraped and pelleted. The cell pellets were lysed in buffer containing 1% NP-40 (Boehringer Mannheim Biochemical, Indianapolis, IN), 1mM EDTA (pH 8.0), 20mM Tris (pH 8.0), 150mM NaCl, 0.15U/ml aprotinin, 10 µg/ml leupeptin, 10 µg/ml pepstatin A and 1mM sodium vanadate, then cleared of insoluble material. Lysates were pre-cleared with protein A agarose (Invitrogen), immunoprecipitated with 8 µg anti-G-CSFR antibody recognizing the N-terminal portion of the G-CSFR (BD PharMingen, San Diego, CA), and analyzed under reducing conditions by SDS-PAGE. The gels were treated with Entensify (DuPont-NEN, Wilmington, DE) according to the manufacturer's instructions and dried. The labeled proteins were visualized by autoradiography.

## Results

### Role of dileucine and tyrosine-based motifs in the G-CSFR in ligand-receptor internalization

Since efficient internalization of many growth factor receptors has been shown to be mediated by dileucine or tyrosine-based motifs in the cytoplasmic domains of these receptors [24]–[26], we investigated whether dileucine and tyrosine-based motifs present in the WT G-CSFR but absent in the Δ716 G-CSFR form might mediate internalization of the G-CSFR complex. Mutations in the G-CSFR corresponding to these motifs were therefore introduced.

**Figure 1:**
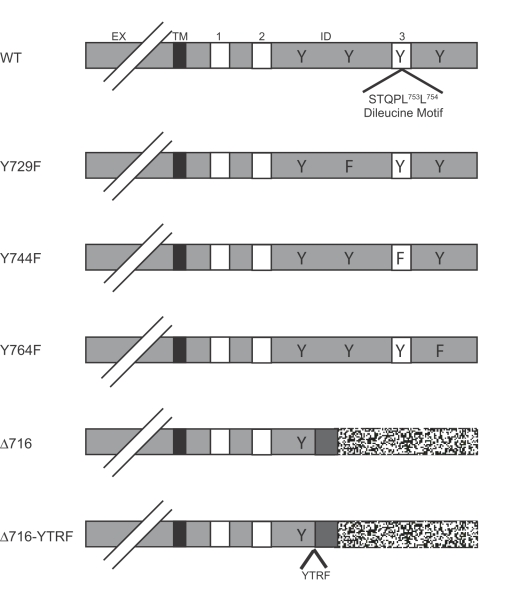
Schematic diagram of G-CSFR forms. The extracellular (EX), transmembrane (TM), and intracellular (ID) domains of the various G-CSFR forms are shown with the conserved box 1, 2, and 3 regions indicated. The full-length wild-type (WT) G-CSFR contains four cytoplasmic tyrosine residues at a.a. positions 704, 729, 744, and 764. The location of the dileucine motif in the WT G-CSFR at residues 749-754, which is deleted in Δ716, where Leu to Ala substitutions were introduced to generate the L753A, L754A, and the L753/754A G-CSFR is shown. Tyr to Phe substituitions at either Tyr^729^, Tyr^744^, or Tyr^764^ were also introduced and the corresponding G-CSFR mutants designated Y729F, Y744F, and Y764F, respectively. The Δ716 G-CSFR was isolated from a patient with SCN/AML and contains a premature stop codon resulting in a truncated G-CSFR. The Δ716 -YTRF mutant was generated by attachment of the transferrin receptor YTRF internalization motif 5′ to the stop codon of the Δ716 G-CSFR.

In previous studies using the Ba/F3 pro-B cell line, and COS-7 cells expressing the WT and truncated G-CSFR forms, we demonstrated that ligand internalization and down-modulation of G-CSFR expression were impaired in cell lines expressing the Δ716 G-CSFR, which is the most common mutant G-CSFR form identified in patients with AML and antecedent SCN [17]. The Δ716 G-CSFR form arises due to a C to T substitution resulting in the generation of a premature stop codon (Figure 1).

To confirm whether our observations of defective internalization and prolonged receptor surface expression in transfected Ba/F3 and COS-7 cells held true in myeloid cells, studies with primary myeloid cells from mice expressing the truncated form of the G-CSFR were done. Similar to our in vitro studies, we observed that G-CSF internalization and down-modulation of the G-CSFR in myeloid cells from the “knock-in” mice are also impaired (data not shown). Since these observations were consistent with our previous in vitro observations [17], additional studies were performed using COS-7 cells.

**Figure 2:**
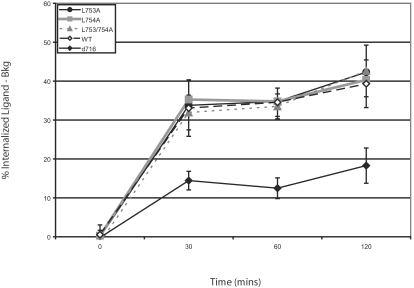
Leucine residues in the STPQLL dileucine motif do not mediate G-CSFR internalization. Mutations in the cytoplasmic dileucine motif of the G-CSFR that is identical to gp130 were introduced by substituting Ala for either Leu^753^ (L753A) or Leu^754^ (L754A) or both Leu^753^ and Leu^754^ (L753/754A). Binding and internalization of [^125^I]-G-CSF were analyzed in COS-7 cells transfected with the WT G-CSFR, Δ716, or G-CSFR forms with Leu to Ala mutations in the dileucine motif. Average data±S.E.M (n = 4) are shown and are expressed as the percent of initial binding at time 0 at 4°C.

The WT G-CSFR contains an STQPLL dileucine motif in its cytoplasmic domain at aa. 749–754 which is identical to the dileucine motif in the cytoplasmic domain of the homologous gp130 molecule which mediates IL-6 internalization and down-modulation of the IL-6R [20], [23]. Both leucine residues in the dileucine motif of gp130 have been shown to be essential for IL-6 internalization [27]. In temperature-shift and acid washing studies with G-CSFR mutants containing either single or double leucine to alanine substitutions at residues 753 and 754, no significant differences in internalized ligand were observed between the WT and L→A transfectants (Figure 2). Ligand internalization was apparent within 30 minutes in cells expressing L753A, L754A, L753/754A, or the WT G-CSFR, with nearly 40% of bound ligand internalized by 30 minutes. Only small increases in internalized ligand were observed at 1 h and 2 h, suggesting ligand internalization is nearly complete by 30 minutes. In contrast, ligand internalization was significantly delayed in the truncated Δ716 G-CSFR (p<0.05 for 30-120 minute time points, compared to WT). These results suggest that unlike the homologous gp130 molecule other cytoplasmic motifs in the G-CSFR mediate ligand/receptor internalization.

### Tyrosine-based motifs in the G-CSFR do not mediate ligand-receptor internalization

**Figure 3:**
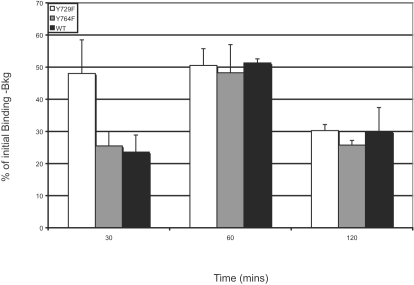
Binding and internalization of [^125^I]-G-CSF in cells transfected with WT, Y729F or Y764F G-CSFR forms. Mutations in the G-CSFR were introduced by subsititution of Phe for either Tyr^729^ (Y729F) or Tyr^764^ (Y764F). COS-7 cells were transfected with each G-CSFR form and ligand binding analyzed. Surface bound ligand was quantitated by acid stripping in 0.5M NaCl (pH 1.0). Internalized ligand was measured after lysis of the cells in 1M NaOH. Data are expressed as a percentage of initial binding at time 0 at 4°C. Values represent the average ±S.E.M (n = 2).

We next investigated the role of tyrosine residues in the cytoplasmic tail of the G-CSFR in mediating receptor internalization. Since Tyr^729^ and Tyr^764^ correspond to known tyrosine-based motifs for other receptors [28] and are deleted in the Δ716 G-CSFR, G-CSFR mutants containing tyrosine to phenylalanine mutations at these locations were generated and designated Y729F and Y764F, respectively. As shown in Figure 3, significant levels of internalized ^125^ I -G-CSF were detected in cells expressing the Y729F and Y764F G-CSFR forms. There was no evidence for defective ligand internalization with the Y729F or Y764F G-CSFR forms compared to the WT G-CSFR over the 2 h time period examined. Although the Y729F receptor mutant exhibited a trend suggesting accelerated internalization compared to the WT receptor form, this was found to be not statistically significant (p = 0.37). These results suggest that loss of either tyrosine residue at position 729 or 764 does not alter ligand/receptor internalization. We also examined the remaining two G-CSFR cytoplasmic tyrosine residues at positions 704 and 744 by introducing tyrosine to phenylalanine mutations and examining ligand internalization in COS-7 cells transduced with these mutant G-CSFR forms, and observed no appreciable alterations in ligand/receptor internalization (data not shown).

### Effect of a C-terminal YTRF internalization motif on internalization and degradation of the Δ716 G-CSFR

**Figure 4:**
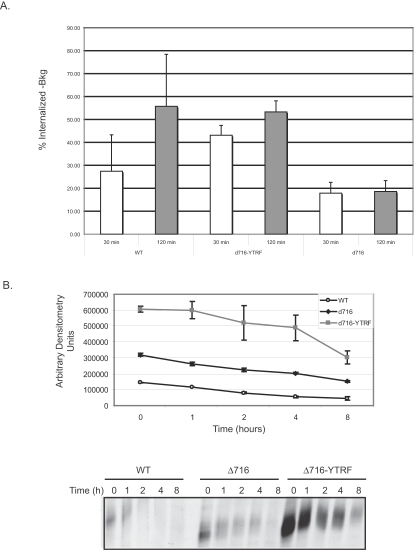
Effect of attachment of an YTRF internalization motif to the Δ716 G-CSFR on ligand/receptor internalization and degradation. (A) Cells expressing the WT, Δ716, or Δ716-YTRF G-CSFR forms were incubated with [^125^I]-labeled G-CSF and subjected to temperature shift and acid washing to determine surface-bound and internalized ligand. Average data±S.E.M (n = 4) are shown as a percentage of initial binding of G-CSF at time 0 at 4°C. (B) Cells expressing the WT, Δ716 or Δ716-YTRF G-CSFR forms were metabolically labeled with [^35^S]-methionine and [^35^S]-cysteine for 1 h, washed then incubated in media containing 1 nM G-CSF and unlabeled amino acids at 37°C. At the indicated times, the cells were lysed, immunoprecipitated with antibody recognizing the N-terminus of the human G-CSFR and analyzed by SDS-PAGE. The blots were subjected to densitometry and the average pixels±S.E.M are shown (n = 2). A representative blot from two independent experiments is shown.

Since our collective data indicated that other sequences in the G-CSFR must regulate ligand/receptor internalization, and since we previously showed that both internalization and degradation of ligand/receptor complexes are impaired in cells expressing the Δ716 G-CSFR, we were interested in determining whether insertion of a known internalization motif into the Δ716 G-CSFR could correct these defects. For these studies, the YTRF internalization motif of the transferrin receptor [23] was inserted in-frame and 5′ to the stop codon of the truncated Δ716 G-CSFR and ligand/receptor internalization and degradation were examined. As shown in Figure 4A, attachment of the YTRF motif corrects the defect in ligand/receptor internalization in cells expressing the Δ716-YTRF G-CSFR. Internalized ^125^I-labeled G-CSF was observed within 30 minutes in cells expressing the WT G-CSFR or the Δ716-YTRF G-CSFR to which the YTRF motif had been attached. At each time point examined, cells expressing the Δ716-YTRF receptor form had significantly higher levels of internalized ligand compared to the Δ716 G-CSFR lacking the fused internalization motif (p<0.01 at each time point).

We next examined whether correction of the internalization defect in Δ716 cells by attachment of the YTRF motif also restored receptor degradation back to normal. We have previously shown that degradation of the WT G-CSFR occurs within 2 h following ligand exposure [17], whereas degradation of the truncated Δ716 G-CSFR is delayed even at 4 h. As shown in Figure 4B, there was no evidence that attachment of the YTRF motif corrected the defect in receptor degradation in cells expressing the Δ716 G-CSFR. The half-life of the WT G-CSFR was determined to be 3.62 h±0.15 compared to 4.84 h±0.11 and 5.9 h±1.02 for Δ716 and Δ716-YTRF, respectively (WT vs. Δ716, p = 0.013 and WT vs. Δ716-YTRF, p = 0.13). No significant difference in receptor half-life was observed between Δ716 and Δ716-YTRF G-CSFR forms (p = 0.24).

## Discussion

SCN is characterized by neutropenia with frequent and often severe bacterial infections [29]. The neutropenia of these patients is generally responsive to pharmacological doses of G-CSF. However, 21% of patients will ultimately develop AML [30]. Mutations in one allele of the gene for the G-CSFR have been identified in 78% of these patients [30], [31]. These mutations introduce a premature stop codon that truncates the cytoplasmic tail of the G-CSFR resulting in hypersensitivity to G-CSF, resistance to apoptosis, and enhanced cell proliferation. Collectively, these observations have led to the postulation that G-CSFR mutations contribute to leukemogenesis in patients with SCN [32].

We and others have previously demonstrated sustained intracellular signaling in response to G-CSF in cells expressing truncated G-CSFR forms isolated from patients with SCN/AML [18], [19], [33]. Our laboratory has also shown that internalization and degradation of the truncated G-CSFR forms is impaired [17]. Thus similar to other human disorders such as Laron dwarfism, type A insulin resistance, and familial hypercholesterolemia in which aberrant down-modulation of growth factor receptors has been shown to play a role in the pathophysiology of these diseases [13]–[16], disruption of receptor trafficking also appears to underlie altered cell signaling in SCN/AML. In this study, we have further investigated the mechanisms that modulate G-CSFR expression and are responsible for G-CSFR internalization and degradation.

For other growth factor receptors, down-modulation of their surface expression has been shown to be mediated by specific amino acid motifs in the receptors themselves and/or involve the recruitment and association of adaptor proteins with the receptors which then target the receptors to intracellular compartments for degradation, recycling, or both [28]. Dileucine motifs in a number of cell surface proteins have been reported to mediate their internalization and sorting in the trans-Golgi apparatus as well as trafficking to the lysosome [24], [26]. Leucine-based internalization motifs have been identified in the mannose 6-phosphate/Insulin –like growth factor receptor, insulin receptor, CD3γ chain, epidermal growth factor receptor, leukemia inhibitory factor receptor, and gp130 subunit of the IL-6 receptor complex [7], [9], [20], [23], [25], [26], [34], [35]. In the gp130 subunit, substitution of the leucine residues in the dileucine STQPLL motif with alanines was shown to dramatically alter trafficking of the IL-6 receptor complex with a near complete loss in the capacity to internalize the receptor complex [20]. In addition, tyrosine containing motifs have also been identified that mediate the internalization of several growth factor receptors, including the receptors for transferrin, low density lipoprotein, IL-2-receptor β chain, and receptors for the insulin-like growth factor (IGF) and mannose 6-phosphate as well as the B-cell receptor α chain,[28], [36]–[40].

Since the identical STQPLL dileucine motif in gp130 that mediates IL-6R internalization is also present at residues 749–754 in the G-CSFR [17], [20], [23], we were interested in determining the role of this motif in internalization of G-CSF/G-CSFR complexes. Notably, this motif is deleted in the Δ716-G-CSFR in patients with SCN/AML. Substitution of either leucine residue or both leucines in this motif in the G-CSFR with alanines had no effect on ligand/receptor internalization, suggesting that the G-CSFR utilizes other mechanisms to regulate its own surface expression. Our results contrast with an earlier report by Aarts *et. al.* demonstrating that mutation of both leucine residues in the STQPLL motif inhibited internalization of the G-CSFR. They reported that mutation of both leucines or the serine residue in the cytoplasmic dileucine motif of the G-CSFR resulted in approximately 50% reduction in receptor endocytosis in 32D cells [41]. The reasons for the discrepancies in our findings and theirs are not clear. It is possible that the differences relate to differences in the cell models used. Since a potential kinase that binds to the dileucine motif to mediate G-CSFR internalization has been postulated [41], it is possible that the unknown kinase is not expressed in COS-7 fibroblast cells. Previous studies with the homologous gp130 molecule of the IL-6R demonstrating a role for the STQPLL motif in receptor internalization utilized a fibroblast model [20], [23]. Hence unlike gp130, the STQPLL dileucine motif does not appear to play a role in regulation of G-CSFR surface expression in a fibroblast model system. The different methods used to analyze receptor internalization may also underlie the discrepancies between our data here and the findings previously reported by Aarts [41]. Aarts' group used flow cytometry to examine internalization, whereas we used more traditional methods employing radiolabeled ligand, temperature shifting, and acid washing.

Our results also demonstrate that Tyr^729^ and Tyr^764^ of the G-CSFR do not contribute to regulation of ligand-induced receptor internalization. Aarts *et. al.* reported that deletion of the dileucine motif including regions upstream or downstream of Tyr^744^ or Tyr^764^ , respectively, resulted in a significant loss of receptor internalization, but deletion of the region surrounding Tyr^729^ did not effect ligand-induced internalization of the receptor [41]. They also reported that a deletion mutant containing the dileucine motif but lacking amino acids surrounding Tyr^764^ failed to properly internalize, and suggested the YXXΦ motif localizing to that region was important in modulating G-CSFR internalization In this study, however, we found that mutation of Tyr^764^ in this motif had no effect, suggesting that other residues in the deletion mutant described by Aarts *et. al.* are important [41]. Notably, in this same region there are several serine residues and a lysine residue. Recently, it has been reported that the lysine residue within the dileucine motif of the CD3γ was important for T-cell receptor (TCR) internalization [42]. It was suggested that there may be a requirement for a positively charged amino acid to facilitate receptor internalization. It is possible that the lysine residue upstream of the YXXΦ motif in the G-CSFR may be important in the proper function of this motif.

As an alternative approach to investigate the mechanisms responsible for the defective receptor internalization and degradation characteristic of SCN/AML cells, we examined the effects of insertion of a known receptor internalization motif into the Δ716 G-CSFR. Attachment of the tyrosine-containing YTRF internalization motif of the transferrin receptor 5′ to the premature stop codon of the Δ716 G-CSFR cDNA was shown to restore receptor internalization back to normal but failed to correct the defect in receptor degradation suggesting that the internalized Δ716 receptor is not properly targeted to the intracellular compartments responsible for degradation of the G-CSFR. Our findings with the Δ716-YTRF G-CSFR suggest that different processes and likely distinct subdomains in the G-CSFR mediate receptor internalization and degradation. We have not examined the potential role of other dileucine motifs in the G-CSFR in mediating receptor internalizations. It is possible that these other dileucine-containing sequences in the G-CSFR may also contribute to G-CSFR internalization.

The use of distinct receptor subdomains by the G-CSFR to regulate these processes would confer an increased capacity for a single chain molecule to “fine tune” signals. Such a mechanism might also be operative in other single chain members of the cytokine receptor superfamily. Further studies of G-CSFR trafficking patterns will help to elucidate the mechanisms responsible for tight regulation of neutrophil numbers within a narrow range in normal hematopoiesis which are disrupted in AML.
